# Interments in the City and Liberties of Philadelphia, from the 31st of July, to the 7th of August

**Published:** 1841-08-14

**Authors:** 


### HEALTH OF THE CITY

Interments *in the City and Liberties of Philadelphia, from the* 31*st of July, to the* 7*th of August.*

| Diseases. | Adults. | Children. |
|---|---|---|
| Apoplexy, | 2 | 0 |
| Congestion of Brains, | 0 | 1 |
| Consumption of the lungs, | 9 | 3 |
| Convulsions, | 1 | 7 |
| Convulsions Puerperal, | 1 | 0 |
| Diarrhœa, | 2 | 5 |
| Dropsy, | 2 | 0 |
| Dropsy of the head, | 0 | 6 |
| —— in the breast, | 3 | 0 |
| Disease of Brain, | 1 | 3 |
| —— Heart, | 2 | 0 |
| —— Liver, | 1 | 0 |
| —— Hip Joint, | 0 | 1 |
| Dysentery, | 5 | 10 |
| Debility | 0 | 6 |
| Enlargement of the Heart, | 1 | 0 |
| Fracture of Leg, | 1 | 0 |
| Fever, | 2 | 0 |
| —— Typhus, | 1 | 2 |
| Gangrene of Bowels, | 1 | 0 |
| Hæmorrhage from Lungs, | 1 | 0 |
| Inflammation of the Brain, | 1 | 6 |
| —— Bronchi, | 0 | 1 |
| —— Lungs, | 0 | 1 |
| —— Stomach, | 0 | 1 |
| —— Bowels, | 3 | 2 |
| | — | — |
| *Carried forward,* | 40 | 56 |

| Diseases. | Adults. | Children. |
|---|---|---|
| *Brought forward,* | 40 | 56 |
| Intemperance, | 1 | 0 |
| Marasmus, | 1 | 10 |
| Measles, | 0 | 1 |
| Morbus Coeruleus, | 0 | 1 |
| Neglect, | 1 | 0 |
| Old age, | 3 | 0 |
| Palsy, | 1 | 0 |
| Softening of Stomach, | 1 | 0 |
| Small pox, | 1 | 3 |
| Still-born, | 0 | 9 |
| Summer Complaint, | 0 | 40 |
| Teething, | 0 | 1 |
| | — | — |
| Total, 170— | 49 | 121 |

Of the above, there were under 1 year, 78

| | |
|---|---|
| From 1 to 2 | 21 |
| 2 to 5 | 7 |
| 5 to 10 | 10 |
| 10 to 15 | 4 |
| 15 to 20 | 1 |
| 20 to 30 | 9 |
| 30 to 40 | 7 |
| 40 to 50 | 9 |
| 50 to 60 | 5 |
| 60 to 70 | 8 |
| 70 to 80 | 7 |
| 80 to 90 | 3 |
| 100 to 110 | 1 |
| Total, | 170 |

Of the above there were 14 from the almshouse, 22 people of colour, and 1 from the country, which are included in the total amount.

